# Correction

**DOI:** 10.1080/20018525.2025.2477431

**Published:** 2025-03-10

**Article title**: Gut microbiota in chronic obstructive pulmonary disease varies by CT-verified emphysema status

**Authors**: Anders Ørskov Rotevatn, Tomas Mikal Eagan, Solveig Tangedal, Gunnar Reksten Husebø, Kristoffer Ostridge and Rune Nielsen

**Journal**: *European Clinical Respiratory Journal*

**Bibliometrics**: Volume 12, Number 01, pages 1-15

**DOI**: https://doi.org/10.1080/20018525.2025.2470499

The authors requested that Figures 2 and 3 be swapped during the proofing stage. However, in the final version, the figures were not swapped correctly. This issue has been addressed, and the corrected version has been republished online.

